# Convergent Evolution of Pathogen Effectors toward Reactive Oxygen Species Signaling Networks in Plants

**DOI:** 10.3389/fpls.2017.01687

**Published:** 2017-09-29

**Authors:** Nam-Soo Jwa, Byung Kook Hwang

**Affiliations:** ^1^Division of Integrative Bioscience and Biotechnology, College of Life Sciences, Sejong University, Seoul, South Korea; ^2^Laboratory of Molecular Plant Pathology, College of Life Sciences and Biotechnology, Korea University, Seoul, South Korea

**Keywords:** pathogen effector, reactive oxygen species, PAMP-triggered immunity, effector-triggered immunity, respiratory burst oxidase homolog, mitogen-activated protein kinase

## Abstract

Microbial pathogens have evolved protein effectors to promote virulence and cause disease in host plants. Pathogen effectors delivered into plant cells suppress plant immune responses and modulate host metabolism to support the infection processes of pathogens. Reactive oxygen species (ROS) act as cellular signaling molecules to trigger plant immune responses, such as pathogen-associated molecular pattern (PAMP)-triggered immunity (PTI) and effector-triggered immunity. In this review, we discuss recent insights into the molecular functions of pathogen effectors that target multiple steps in the ROS signaling pathway in plants. The perception of PAMPs by pattern recognition receptors leads to the rapid and strong production of ROS through activation of NADPH oxidase Respiratory Burst Oxidase Homologs (RBOHs) as well as peroxidases. Specific pathogen effectors directly or indirectly interact with plant nucleotide-binding leucine-rich repeat receptors to induce ROS production and the hypersensitive response in plant cells. By contrast, virulent pathogens possess effectors capable of suppressing plant ROS bursts in different ways during infection. PAMP-triggered ROS bursts are suppressed by pathogen effectors that target mitogen-activated protein kinase cascades. Moreover, pathogen effectors target vesicle trafficking or metabolic priming, leading to the suppression of ROS production. Secreted pathogen effectors block the metabolic coenzyme NADP-malic enzyme, inhibiting the transfer of electrons to the NADPH oxidases (RBOHs) responsible for ROS generation. Collectively, pathogen effectors may have evolved to converge on a common host protein network to suppress the common plant immune system, including the ROS burst and cell death response in plants.

## Introduction

Plants have evolved sophisticated defense mechanisms to resist potential attacks by microbial pathogens (Grant and Loake, 2000). The first line of defense is triggered in plants by the perception of microbe- or pathogen-associated molecular patterns (MAMPs or PAMPs) via membrane-bound pattern recognition receptors (PRRs), leading to basal immunity, known as PAMP-triggered immunity (PTI) (Gómez-Gómez and Boller, 2000; Jones and Dangl, 2006). PAMPs include conserved cell surface structures including bacterial flagellin, lipopolysaccharides, and peptidoglycan, or fungal cell wall components such as glucan or chitin (Zipfel et al., 2004; Torres, 2010). Plants may disrupt numerous non-host or host pathogen attacks via PTI; however, adapted pathogens can overcome the PTI-dependent defense response to cause disease on their host plants (Collins et al., 2003; He et al., 2006). PTI requires signal transduction from receptors to downstream components via mitogen-activated protein kinase (MAPK) cascade pathways (Pitzschke et al., 2009). Known PAMPs activate MAP kinases in plant cells. In the second line of defense, plants have acquired a cell-based surveillance system using intracellular nucleotide-binding leucine-rich repeat (NLR) receptors to recognize specific pathogen effectors, leading to resistance (*R*) gene-mediated effector-triggered immunity (ETI) (Jones and Dangl, 2006; Dangl et al., 2013). The two phases of plant immunity may be spatiotemporally distinct but are intimately related to the reactive oxygen species (ROS) burst (Grant and Loake, 2000; Torres et al., 2006; Kadota et al., 2015). Production of ROS in plant cells is a hallmark of successful recognition of plant pathogens and activation of plant defenses (Lamb and Dixon, 1997; Torres, 2010). Pathogen-induced apoplastic ROS production was first demonstrated in potato tuber tissues by Doke () and indeed ROS play important roles in plant immune responses as signaling molecules (Torres, 2010; Mittler et al., 2011; O’Brien et al., 2012a; Frederickson Matika and Loake, 2014; Lehmann et al., 2015). In plant cells, ROS also occur in response to many physiological stimuli (Mori and Schroeder, 2004; Mittler et al., 2011; Baxter et al., 2014).

**FIGURE 1:**
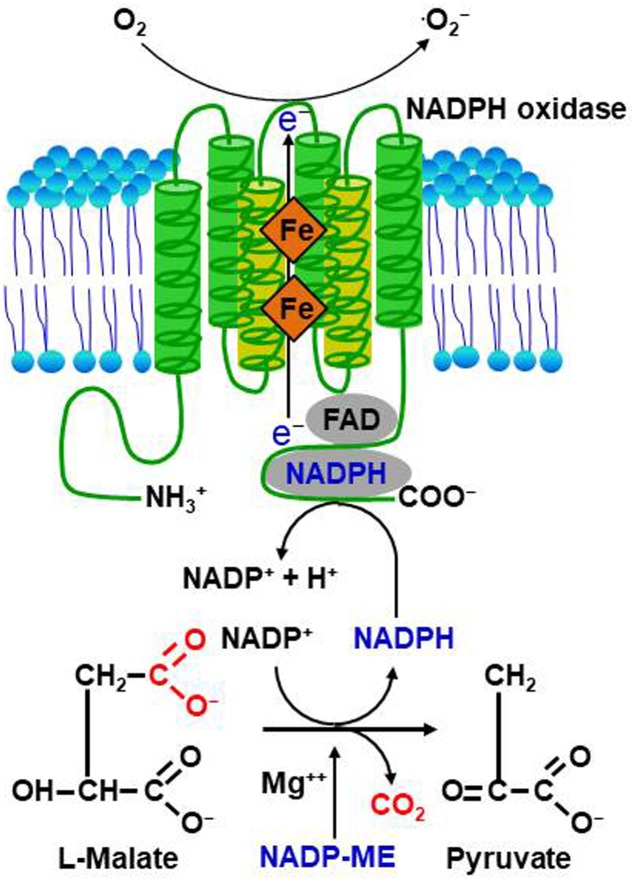
Proposed model for NADPH oxidase-dependent ROS generation via NADP-malic enzyme (ME)-mediated electron supply. NADP-malic enzyme (ME) serves as a source of NADPH and pyruvate in the cytosol of various plant tissues. It catalyzes the oxidative decarboxylation of L-malate to yield pyruvate, CO_2_, and NADPH in the presence of a bivalent cation, such as Mg^++^. NADPH oxidase, known as the Respiratory Burst Oxidase Homolog (RBOH), catalyzes the generation of superoxide (⋅O_2_^-^) by the one-electron reduction of molecular oxygen using NADPH as an electron donor. Superoxide can spontaneously form hydrogen peroxide (H_2_O_2_) that will undergo further reactions to generate reactive oxygen species (ROS).

Reactive oxygen species are highly reactive reduced oxygen molecules, such as superoxide (⋅O_2_^-^), hydrogen peroxide (H_2_O_2_), and hydroxyl radical (⋅OH) (Grant and Loake, 2000; Mori and Schroeder, 2004; Sagi and Fluhr, 2006). There is comprehensive evidence that ROS can act as cellular signaling molecules, mediating various important responses of plant cells to different physiological stimuli, including pathogen attack, abiotic stress, hormone signaling, and polar growth (Mori and Schroeder, 2004; Torres and Dangl, 2005). ROS are formed intracellularly during certain redox reactions in the cell membranes, cytoplasm, nuclei, mitochondria, chloroplasts, peroxisomes, and endoplasmic reticulum (Ashtamker et al., 2007; Liu et al., 2007; Torres, 2010; La and López-Huertas, 2016). The endomembrane and nuclear compartments are likely targets or sources for ROS signaling (Ashtamker et al., 2007). Pathogen-induced ROS generation in chloroplasts is known to play a crucial role in the signaling for and/or execution of hypersensitive response (HR) cell death in plants (Liu et al., 2007). In addition, mitochondrial ROS associated with alteration in respiration are likely to activate defense responses, but could not be directly involved in plant cell death (Vidal et al., 2007). The generation of extracellular ROS, including H_2_O_2_, requires the extracellular activities of cell wall peroxidases and plasma membrane NADPH oxidases in plant cells (Bienert and Chaumont, 2014). NADPH oxidase-dependent ROS generation with electron supply provided by NADP-malic enzyme (ME) is presented in **Figure 1**. H_2_O_2_ is one of the most abundant and stable ROS in plants, and ROS generated inside cells can move apoplastically as H_2_O_2_ into neighboring cells (Allan and Fluhr, 1997). ROS signal transduction activates Ca^2+^ channels in the plasma membrane and may be a central step in many ROS-mediated processes regulating the physiology of plant cells (Mori and Schroeder, 2004). It has also been proposed that biomembrane channels (aquaporins) mediate H_2_O_2_ transport across biological membranes to control ROS signaling in plant cells (Bienert and Chaumont, 2014; Tian et al., 2016). The primary ROS bursts after perception of pathogens occur in the apoplasts; however, ROS produced in different compartments inside the plant cell may function in plant defenses to pathogen invasion (Torres, 2010).

Reactive oxygen species are an effective weapon that can be produced rapidly and utilized against pathogen infection (Levine et al., 1994). H_2_O_2_ and O_2_^-^ are mainly produced at the site of attempted pathogen invasion in plant cells (Apostol et al., 1989). They are secreted from the cell within 3 min of recognition of MAMPs or PAMPs (Chinchilla et al., 2007; Nühse et al., 2007; Hedrich, 2012). The function of H_2_O_2_ in inhibiting pathogen growth is fully understood (Chi et al., 2009; Huang et al., 2011; Park et al., 2013). Plant-derived ROS act as a powerful weapon against pathogen invasion. By contrast, pathogens have evolved strategies to reduce plant ROS bursts in different ways during infection. For example, pathogens may avoid the risk of the ROS burst by using effectors. However, in the presence of effector-aware and defensible plants, pathogens are often faced with mutation pressure for virulence (Jones and Dangl, 2006). The competition between pathogens and host plants, called ‘arms races’ (Boller and He, 2009), may lead to the creation of numerous types of effectors in microbial pathogens and also R- or defense-related proteins in plants. Effectors from these evolutionarily diverse pathogens are predicted to converge on common host plant proteins, which are characterized by a high degree of interaction in host plant protein networks (Weßling et al., 2014; Rovenich et al., 2016). Here, we review and discuss recent advances in understanding how microbial pathogen effectors have evolved toward the suppression of plant apoplastic ROS bursts during infection.

## Pamp-Mediated ROS Burst that Triggers Basal Immune Responses

**FIGURE 2:**
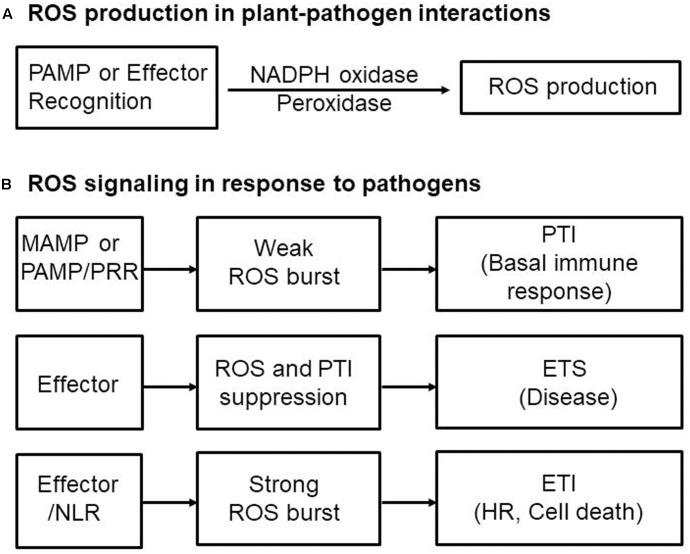
Reactive oxygen species (ROS) production and signaling in plant-pathogen interactions. **(A)** Perception of microbe- or pathogen-associated molecular patterns (MAMPs or PAMPs) or effectors by plants via membrane-bound pattern recognition receptors (PRRs) or intracellular nucleotide-binding leucine-rich repeat (NLR) receptors, respectively, activates NADPH oxidases as well as peroxidases, triggering ROS production and the ROS burst. **(B)** Perception of MAMPs or PAMPs by PRRs induces weak ROS bursts, leading to PAMP-triggered immunity (PTI)-dependent basal defense responses. Adapted pathogens secrete virulent effector proteins into plant cells to suppress the ROS burst and PTI, resulting in effector-triggered susceptibility (ETS) to cause disease in their respective host plants. Pathogen avirulence (Avr) effectors interact directly or indirectly with intracellular NLR proteins, leading to a strong ROS burst and HR cell death response, key components of effector-triggered immunity (ETI). ETI is an accelerated and amplified PTI response, resulting in disease resistance and, usually, a resistance (*R*) gene-mediated HR cell death response at the infection site in plants.

Perception of PAMPs by plants via PRRs triggers ROS production through activation of NADPH oxidases as well as peroxidases, leading to PTI-dependent basal defenses that inhibit invading pathogens (**Figure 2**). The apoplastic ROS bursts generated in elicited plant cells are sufficiently cytotoxic to kill invading pathogens (Legendre et al., 1993; Chi et al., 2009; Park et al., 2013). ROS also act as signaling molecules, triggering plant immune and cell death responses (Tenhaken et al., 1995; Jabs, 1999; Torres, 2010). Thus, pathogens need to take steps to avoid exposure to toxic ROS. NADPH oxidases, also known as Respiratory Burst Oxidase Homologs (RBOHs), are responsible for production of ROS in plants during pathogen infection (Torres and Dangl, 2005; Torres, 2010). NADPH oxidase (RBOHD) phosphorylation by the PRR-associated kinase BIK1 has been proposed to be essential for PAMP-triggered ROS production (Kadota et al., 2014). In addition, the apoplastic peroxidase-dependent ROS burst plays an important role in *Arabidopsis* PTI mediated by the recognition of PAMPs (Daudi et al., 2012). Antisense expression of a heterologous French bean (*Phaseolus vulgaris*) peroxidase (*FBP1*) cDNA in *Arabidopsis* diminishes the expression of *Arabidopsis* peroxidases PRX33 and PRX34, blocking the ROS burst in response to a *Fusarium oxysporum* cell wall elicitor, and leading to enhanced susceptibility to fungal and bacterial pathogens (Bindschedler et al., 2006; Daudi et al., 2012; O’Brien et al., 2012b). Similarly, pepper extracellular peroxidase CaPO2 generates ROS bursts, activating local and systemic cell death and defense response to bacterial pathogens (Choi et al., 2007). Recently, the plant aquaporin AtPIP;4 has been demonstrated to trigger cytoplasmic import of apoplastic H_2_O_2_ into plant cells, activating systemic acquired resistance (SAR) and PTI pathways in response to *Pseudomonas syringae* pv. *tomato* DC3000 and two typical PAMPs (flagellin and chitin), respectively (Tian et al., 2016). This suggests a pivotal role for aquaporins in apocytoplastic ROS signal transduction in disease immunity pathways.

Plants defend themselves against invading pathogens through cell wall reinforcement. Cell wall fortifications are facilitated by an apoplastic H_2_O_2_ burst, cell wall cross-linking, and callose deposition at the site of infection (Bradley et al., 1992; Deepak et al., 2007; Luna et al., 2011; Ellinger et al., 2013). Effector (Elicitor)-induced oxidative cross-linking of plant cell wall structural proteins is essential for cell maturation and toughening of cell walls in the initial stages of plant defense (Bradley et al., 1992). Callose, a (1,3)-β-glucan, is a major component of cell wall thickening at sites of fungal penetration in plants (Ellinger et al., 2013). The *Arabidopsis* GTPase RabA4c physically interacts with its effector PMR4 to enhance PMR4-dependent callose biosynthesis, which ultimately results in complete penetration resistance to powdery mildew (*Golovinomyces cichoracearum*) (Ellinger et al., 2014). Hydroxyproline-rich glycoproteins (HRGPs) are involved in cell wall strengthening by formation of intra- and intermolecular cross-links (Deepak et al., 2007). Exogenous application of H_2_O_2_ rescues the callose deposition-deficient phenotype of peroxidase knockdown *Arabidopsis* lines treated with the bacterial flagellin, Flg22 (Daudi et al., 2012). This suggests that cell wall peroxidase-dependent H_2_O_2_ production is required for PAMP-triggered immune responses, such as callose deposition.

## Effector-Mediated ROS Burst that Induces HR and Cell Death Responses

Reactive oxygen species bursts are monitored during infection by avirulent pathogens (Grant and Loake, 2000). Pathogen avirulence (Avr) effectors interact directly or indirectly with NLR proteins, leading to a strong ROS burst and the HR cell death response, both key components of ETI (**Figure 2**; Gabriel and Rolfe, 1990; McHale et al., 2006; van der Hoorn and Kamoun, 2008; Spoel and Dong, 2012; Cesari et al., 2014; Han and Hwang, 2017). However, whether NLR-mediated ROS themselves activate HR cell death and immune responses is not fully understood. Rice resistance to incompatible rice blast fungus (*Magnaporthe oryzae*) isolates is suppressed by inhibiting the accumulation of apoplastic ROS, even in the presence of the *R* gene (Singh et al., 2016). The resistant rice cultivar Hwayeonbyeo carrying *Pib* exhibits a compatible response to *M. oryzae* INA168 carrying *AvrPii* and *AvrPib* via the deletion of *OsNADP-ME2.* In the absence of normal production of ROS, cell death and immune responses are severely suppressed, although the Avr effector and cognate NLR proteins are not impaired (Singh et al., 2016). Increased ROS production during infection is essential for NLR-mediated cell death and immunity as well as disease-associated cell death (Greenberg and Yao, 2004; Choi et al., 2013). Perception of plant NLR receptors by specific pathogen effectors triggers a strong ROS burst through activation of RBOHs receiving an adequate supply of NADPH via the activity of NADP-ME (**Figure 1**; Singh et al., 2016).

Host cysteine proteases targeted by the *Ustilago maydis* effector Pit2 are likely to be crucial determinants in apoplastic maize immune responses such as the ROS burst (Mueller et al., 2013). The *Cladosporium fulvum* effector Avr2 binds and inhibits Rcr3, a extracellular tomato cysteine protease, which is required for *Cf-2*-dependent disease resistance (Rooney et al., 2005). It has been proposed that the Rcr3-Avr2 complex enables the Cf-2 protein to activate a HR, including the ROS burst and cell death (**Table 1**). The defense-related protease Rcr3 may act as a decoy for Avr2 perception in tomato plants carrying the *Cf-2* resistance gene (Shabab et al., 2008).

**Table 1:** Effector targets associated with ROS signaling in plants.

Effector	Pathogen	Host	Target	Reference
**Plant–pathogen interface**				
Pep1	*Ustilago maydis*	Maize	Apoplastic peroxidase	Hemetsberger et al., 2012
Catalase B	*Blumeria graminis*	Barley	Apoplastic H_2_O_2_	Zhang et al., 2004
Avr4	*Cladosporium fulvum*	Tomato	Fungal cell wall (Chitin)	van den Burg et al., 2006
Mg3LysM	*Mycosphaerella graminicola*	Wheat	Fungal cell wall (Chitin)	Marshall et al., 2011
Pit2	*U. maydis*	Maize	Cysteine protease	Mueller et al., 2013
Avr2	*Cladosporium fulvum*	Tomato	Cysteine protease Rcr3	Shabab et al., 2008
**MAPK signaling pathway**				
AvrPtoB	*Pseudomonas syringae* pv. *tomato*	Arabidopsis	LysM receptor kinase	Gimenez-Ibanez et al., 2009
HopAO1	*P. syringae* pv. *tomato*	Arabidopsis	EF-TU RECEPTOR	Macho et al., 2014
Avr Pto	*P. syringae* pv. *tomato*	Arabidopsis	BAK1	Shan et al., 2008
HopF2	*P. syringae* pv. *tomato*	Arabidopsis	BAK1, MAPKs	Wu et al., 2011; Zhou et al., 2014
HopF2	*P. syringae* pv. *tomato*	Arabidopsis	MKK5	Wang et al., 2010
HopAI1	*P. syringae* pv. *tomato*	Arabidopsis	MAPKs	Zhang et al., 2007, 2012
PopP2	*Ralstonia solanacearum*	Arabidopsis	WRKY TFs	Le Roux et al., 2015
AvrAC	*Xanthomonas campestris* pv. *campestris*	Arabidopsis	BIK1	Feng et al., 2012
**Vesicle trafficking**				
HopM1	*P. syringae* pv. *tomato*	Arabidopsis	AtMIN7	Nomura et al., 2006, 2011
Cryptogein	*Phytophthora cryptogea*	Tobacco	RBOHD	Noirot et al., 2014
**Metabolic priming**				
AvrPii	*Magnaporthe oryzae*	Rice	NADP-malic enzyme2	Singh et al., 2016
AvrBsT	*X. c.* pv. *vesicatoria*	Pepper	Arginine decarboxylase	Kim et al., 2013
AvrRxo1	*X. oryzae* pv. *oryzicola*	Rice	NAD	Shidore et al., 2017

The *Xanthomonas campestris* pv. *vesicatoria* effector AvrBsT induces a H_2_O_2_ burst and HR cell death in pepper (*Capsicum annuum*) (Kim et al., 2013). The AvrBsT-triggered HR cell death response is similar to the resistance (*R*) gene-mediated defense response in plants (Eitas and Dangl, 2010; Kim et al., 2010). AvrBsT physically binds to pepper arginine decarboxylase (CaADC1) (Kim et al., 2013), pepper aldehyde dehydrogenase (CaALDH1) (Kim and Hwang, 2015a), pepper heat shock protein 70a (CaHSP70a) (Kim and Hwang, 2015b), and pepper suppressor of the G2 allele of skp1 (CaSGT1) (Kim et al., 2014) *in planta* to promote the ROS burst, defense gene expression, cell death, and defense responses (Han and Hwang, 2017). AvrBsT and CaPIK1 directly bind to CaSGT1 in yeast and *in planta*. AvrBsT is subsequently phosphorylated by CaPIK1 and forms the active AvrBsT–SGT1–SGT1-PIK1 complex, which promotes the ROS burst, HR cell death, and defense responses in plants (Kim et al., 2014).

## Effector-Mediated ROS Suppression that Causes Disease on Host Plants

Adapted microbial pathogens have evolved their effector proteins as virulence factors to suppress the ROS burst and PTI, causing disease on their respective host plants (**Figure 2**). Secreted effector proteins are delivered into host cells to protect pathogen cell walls against plant-derived hydrolytic enzymes and suppress PAMP-triggered host immunity, leading ultimately to the successful colonization of host plants (de Jonge et al., 2010; Mentlak et al., 2012; Lee et al., 2014; Sánchez-Vallet et al., 2014; Wawra et al., 2016). Various components of fungal cell walls such as glucans, chitin, and proteins, acting as PAMPs to trigger basal immune responses, are degraded by plant-derived hydrolytic enzymes, such as β-1,3-glucanases, chitinases, and serine and cysteine proteases (Jones and Dangl, 2006; van den Burg et al., 2006).

The *C. fulvum* effector Avr4, a chitin-binding lectin, binds to its own cell walls to protect chitin against hydrolysis by plant chitinases during infection of tomato, suggesting that Avr4 is a virulence factor (**Table 1**; van den Burg et al., 2006). The LysM domain-containing effector protein Ecp6 of *C. fulvum* mediates virulence through suppression of chitin-triggered immunity in plants (de Jonge et al., 2010). *C. fulvum* Ecp6 (CfEcp6) is secreted at high levels during plant infection and binds chitin, thereby blocking chitin-triggered immunity responses through sequestering chitin fragments and preventing their recognition by plant chitin receptors. By contrast to *C. fulvum* Ecp6, both Mg1LysM and Mg3LysM from the fungus *Mycosphaerella graminicola* protect fungal hyphae against plant-derived hydrolytic enzymes, such as chitinases (**Table 1**; Marshall et al., 2011). As a virulence determinant in the rice blast fungus *M. oryzae*, the secreted LysM effector (Slp1) binds to chitin inside the fungal cell wall, suppressing chitin-triggered plant immune responses, including the ROS burst and plant defense gene expression (Mentlak et al., 2012). The effector Slp1 inhibits the chitin-induced ROS burst in rice suspension cells. The lectin FGB1 (Fungal Glucan Binding 1), secreted from the root endophyte *Piriformospora indica*, specifically interacts with β-1, 6-linked glucan, altering cell wall composition and suppressing glucan-triggered ROS production in plants (Wawra et al., 2016). The presence of *P. indica* in the roots of barley inhibits laminarin-induced ROS production. Laminarin-triggered ROS production is also delayed when compared with that observed following chitin elicitation.

Some pathogen effectors specifically bind to plant proteases and may activate downstream signaling components (van der Hoorn and Jones, 2004). Apoplast-localized plant proteases can play an important role in defense responses to microbial pathogens. The *U. maydis* effector Pit2 physically interacts with and inhibits apoplastic maize cysteine proteases, suppressing host immunity (**Table 1**; Mueller et al., 2013). The secreted effector protein Pit2 is essential for maintenance of biotrophy and induction of tumors in maize. The biotrophic interaction of maize with *U. maydis* depends on inhibition of apoplastic cysteine proteases by the effector Pit2 with a conserved inhibitor domain. The *U. maydis* effector Pep1 (Protein essential during penetration-1) interferes with maize apoplastic peroxidases at the plant–pathogen interface to scavenge ROS (**Table 1**; Hemetsberger et al., 2012). As an inhibitor of plant peroxidases, Pep1 effectively inhibits the peroxidase-triggered ROS burst, which thereby suppresses the early immune response of maize leading to the establishment of a biotrophic interaction. Pep1 localizes to the plant apoplast, where it accumulates at sites of cell-to-cell passage of biotrophic *U. maydis* hyphae (Doehlemann et al., 2009). The obligate biotrophic fungal pathogen of barley, *Blumeria graminis* f. sp. *hordei*, secretes an extracellular catalase B to scavenge H_2_O_2_ at sites of fungal germ tube invasion during infection (**Table 1**; Zhang et al., 2004). A large number of *cat*B transcripts accumulate during the mature primary germ tube and appressorium germ tube stages of fungal development on the susceptible barley plant, suggesting the upregulation of an extracellular catalase gene early during fungal invasion.

## Effector-Mediated ROS Suppression that Targets MAPK Signaling Pathways

**FIGURE 3:**
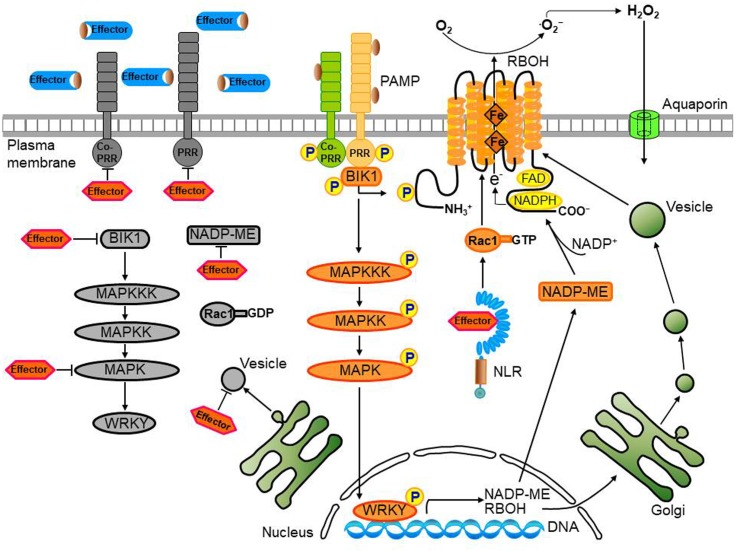
Convergent effector targeting of ROS signaling networks in plant cells. Plant pathogens secrete effectors into the apoplastic area and cytoplasm of plant cells. These apoplastic effectors interfere with PRR-mediated PAMP recognition, ultimately leading to the inactivation of plasma membrane-bound RBOHs. Cytoplasmic effectors target the kinase domains of PRRs, the receptor-like cytoplasmic kinase (RLCK) BIK1, MAPK cascades, and WRKY transcription factors, inhibiting the transcription of *RBOHs* and *NADP-ME*, both essential for robust ROS generation. A bacterial type III effector inhibits BIK1 downstream of the PRR (BAK1), enhancing virulence. Some cytoplasmic effectors target vesicle trafficking, suppressing the transport of ROS-producing RBOH enzymes to the plasma membrane during infection. Secreted pathogen effectors block the metabolic coenzyme NADP-ME, inhibiting the transfer of electrons to the NADPH oxidases (RBOHs) responsible for ROS generation. The biomembrane channels, aquaporins, mediate H_2_O_2_ transport across biological membranes. However, when specific pathogen effectors recognize intracellular NLR proteins, the effector no longer functions as a virulence factor; here, the effector-NLR complex leads to a strong apoplastic ROS burst and HR cell death response. The PRR-associated kinase BIK1 directly phosphorylates the NADPH oxidase RBOHD to enhance RBOHD-mediated ROS production. Rac1 directly interacts with both NLR and RBOH to activate RBOH. Apoplastic ROS are toxic to pathogens and also activate MAPK cascades and RBOH enzymes in their role as immune signal molecules.

During pathogen infection, perception of PRRs by PAMPs leads to PTI by causing a rapid ROS burst via receptor-like cytoplasmic kinases (RLCKs) such as PBL1 and BIK1 (Feng et al., 2012; Shi et al., 2013; Ranf et al., 2014) or through MAPK cascades (Zhou et al., 2014). Pathogens overcome basal immune responses through inactivation of PAMP-induced signaling pathways that target MAPK cascade components (Pitzschke et al., 2009; Bi and Zhou, 2017). MAPKs are major targets for inactivation by pathogen effector proteins (**Figure 3**). MAPK cascades are highly conserved modules and are implicated in pathogen signaling during multiple defense responses against pathogen invasion in plants (Yang et al., 2001; Colcombet and Hirt, 2008; Tena et al., 2011; Singh et al., 2012, 2014, 2016). In particular, the MAPK cascade regulates transcriptional reprogramming via the WRKY transcription factor in the early signaling events following PAMP recognition in plants (Adachi et al., 2015). MAPK signaling is involved in the expression of genes required for apoplastic ROS production in plant defense responses. For example, WRKY transcription factors phosphorylated by MAPKs upregulate the RBOH, an NADPH oxidase, inducing pathogen-responsive ROS bursts in *Nicotiana benthamiana* (Adachi et al., 2015). However, because of the complicated nature of the downstream MAPK cascade, how MAPK signaling promotes ROS generation is not fully understood.

Pathogen effectors act on plant host target proteins, interfering with PTI-mediated defense signaling cascades, such as ROS and MAPK cascades, ultimately causing disease in host cells (Jones and Dangl, 2006). For example, the *P. syringae* effector HopAO1 targets the *Arabidopsis* receptor kinase EF-TU RECEPTOR (EFR), reducing EFR phosphorylation, thereby preventing subsequent early immune responses, such as the ROS burst and MAPK activation (**Table 1**; Macho et al., 2014). The *P. syringae* effector HopF2 interacts directly with the plasma membrane-localized receptor-like kinase (RLK) BAK1 and suppresses early signaling events triggered by multiple PAMPs, including BIK1 phosphorylation, MAPK activation, and defense gene expression (**Table 1**; Wu et al., 2011; Zhou et al., 2014). In fact, BAK1 can directly phosphorylate the plasma membrane-localized RLCK BIK1 (Lu et al., 2010). FLAGELLIN SENSING2 (FLS2)/BAK1-induced MAPK signaling (Zhou et al., 2014) enhances gene expression for plant immune responses via recognition of specific target genes by WRKY transcription factors (Chi et al., 2013). Multiple WRKYs bind to and activate the NADPH oxidase RBOHB promoter, followed by enhanced *RBOHB* expression, which subsequently leads to the RBOHB-dependent ROS burst (Adachi et al., 2015). Type III effectors also target specific host plant proteins, such as major MAPK and WRKY modules (Feng and Zhou, 2012; Le Roux et al., 2015). The *P. syringae* effector HopAI1, for example, inactivates MAPKs by removing the phosphate group from phosphothreonine through a unique phosphothreonine lyase activity (**Table 1**; Zhang et al., 2007, 2012). Another type III effector, HopF2, interacts with *Arabidopsis* MAP Kinase Kinase 5 (MKK5), and likely other MAPKKs (MKK1, MKK3, MKK4, MKK6, and MKK10), suppressing MAPKs and PAMP-triggered defenses (**Table 1**; Wang et al., 2010). The *Ralstonia solanacearum* acetyltransferase effector PopP2 localizes to the plant cell nucleus and acetylates lysine residues of WRKY transcription factors, blocking DNA binding (**Table 1**; Deslandes et al., 2003;Le Roux et al., 2015). The *X. campestris* uridylyl transferase effector AvrAC targets and inhibits BIK1 downstream of BAK1, enhancing virulence (**Table 1**; Feng et al., 2012). In particular, AvrAC acts upstream of MAPK cascades and ROS production to suppress PTI signaling. The PRR-associated kinase BIK1 directly phosphorylates the NADPH oxidase RBOHD, enhancing RBOHD-mediated ROS production (**Figure 3**; Kadota et al., 2014; Li et al., 2014). The *Phytophthora infestans* RXLR effector PexRD2 interacts with a specific host MAPKKK, suppressing MAPKKK signaling-dependent cell death (King et al., 2014). In addition, multiple RXLR effectors from *Hyaloperonospora arabidopsidis* and *P. infestans* suppress the PAMP-elicited ROS burst (Fabro et al., 2011; Zheng et al., 2014). Functionally redundant effectors from different pathogen species may evolve virulence strategies to target PTI signal transduction processes, such as MAPK cascades, the ROS burst, and defense gene activation (**Figure 3**; Kvitko et al., 2009; Win et al., 2012; Zheng et al., 2014).

## Effector-Mediated ROS Suppression that Targets Vesicle Trafficking and Metabolic Priming

Vesicle trafficking is an important cellular function in plants and is required for the transport of immune receptors and associated proteins, and for the extracellular secretion of immune-related molecules and antimicrobial compounds upon pathogen attack (Inada and Ueda, 2014; Macho and Zipfel, 2015). Visible vesicle-like bodies aggregate directly beneath sites of fungal attack in the barley-*B. graminis* f. sp. *hordei* pathosystem (An et al., 2006) and vesicle incidence is positively associated with levels of resistance to *B. graminis* f. sp. *hordei* penetration (Collins et al., 2003). Vesicles contain phytoalexins, phenolics, or ROS (Hückelhoven et al., 1999; Collins et al., 2003; An et al., 2006). The presence of ROS in the vesicles at penetration sites of *B. graminis* f. sp. *hordei* (Collins et al., 2003) suggests that the endomembrane-associated immune response is associated with ROS signaling. One constituent of the vesicles is H_2_O_2_, a plant defense compound involved in antimicrobial, cell wall cross-linking, and signaling functions (Lamb and Dixon, 1997; Collins et al., 2003).

Pathogen effectors target vesicle trafficking, suppressing PTI and potentially mediating immune-related ROS signaling (**Figure 3**; Macho and Zipfel, 2015). The *Phytophthora cryptogea* effector cryptogein induces an increase in abundance of the ROS-producing enzyme RBOHD, as well as ROS production at the plasma membrane of tobacco cells (**Table 1**; Noirot et al., 2014). Plant NADPH oxidases (RBOHDs) localize to the plasma membrane and endomembranes, and vesicle trafficking may contribute to the increase in RBOH abundance at the plasma membrane. The *P. syringae* pv. *tomato* virulence effector HopM1 interacts with and degrades an immunity-associated protein AtMIN7 via the host proteasome, suppressing PTI (**Table 1**; Nomura et al., 2006). AtMIN7 localizes to the *trans*-Golgi network/early endosome of plant cells and mediates immune-associated vesicle trafficking (Nomura et al., 2011). Some type III effector proteins from *X. campestris* pv. *vesicatoria* (*Xcv*) target plant protein secretion pathways, suppressing PTI (Macho and Zipfel, 2015). The *Xcv* type III effector XopJ, a member of the YopJ family of SUMO peptidases and acetyltransferases, is attached to the plasma membrane of plant cells through a myristoylation motif and interferes with host-cell protein secretion, inhibiting immune-associated callose deposition at the cell wall (Bartetzko et al., 2009; Üstün et al., 2013). In addition, XopB and XopS contribute to *Xcv* virulence, suppressing PAMP-triggered gene expression (Schulze et al., 2012). XopB localizes to the Golgi vesicles and cytoplasm and interferes with plant cell protein secretion (Schulze et al., 2012). However, specific PTI-related targets of these *Xcv* effectors are unknown. Overall, better understanding of the role of vesicle trafficking in promoting plant immune responses related to ROS signaling requires further experimental evidence.

Metabolic priming by secreted pathogen effectors has emerged as a common strategy for host manipulation (**Figure 3**). *U. maydis* secretes chorismate mutase (Cmu1) as a virulence factor into plant cells, suppressing plant defense responses associated with pathogen-induced salicylic acid biosynthesis (Djamei et al., 2011). During *U. maydis* infection in maize, Cmu1 is translocated into plant cells, spreads to neighboring cells, and can change the metabolic status of plant cells through metabolic priming. Pathogen effectors may target plant enzymes required for ROS production in the key metabolic pathways. For instance, the chloroplastic enzyme aspartate oxidase, which is involved in NAD metabolism, is required for the NADPH oxidase RBOHD-mediated ROS burst that is triggered by the perception of several unrelated PAMPs (Macho et al., 2012). Notably, inducible NAD overproduction in *Arabidopsis* transcriptionally up-regulates aspartate oxidase during the incompatible infection with *P. syringae* pv. *tomato* (*avrRpm1*) (Pétriacq et al., 2012). Intracellular NAD acts as an integral regulator of multiple defense layers to trigger the production of ROS and defense hormones (Pétriacq et al., 2016). Manipulation of abscisic acid (ABA) content in *Arabidopsis* modulates ROS production via the control of peroxidase activity in response to *Dickeya dadantii* infection (van Gijsegem et al., 2017). Increased ABA contents seem likely to correlate with reduced ROS production and with enhanced disease susceptibility. The *M. oryzae* effector AvrPii specifically interacts with rice NADP-malic enzyme2 (OsNADP-ME2) to suppress the host ROS burst (**Table 1**; Singh et al., 2016). Indeed, purified AvrPii proteins inhibit *in vitro* NADP-ME activity. NADP-ME, also known as a coenzyme, catalyzes the oxidative decarboxylation of malate and NADP^+^, and provides NADPH as an electron donor for plasma membrane-bound NADPH oxidase (**Figure 1**), which is essential for the apoplastic ROS burst at the infection site (Doubnerová and Ryšlavá, 2011). NADP-ME is involved in the production of ROS during early plant basal defense against the hemi-biotrophic fungal pathogen *Colletotrichum higginsianum* (Voll et al., 2012). The *X. oryzae* pv. *oryzicola* effector AvrRxo1 targets and phosphorylates the central metabolite and redox carrier NAD *in planta*, and this catalytic activity is required for suppression of the ROS burst (Shidore et al., 2017). The *X. campestris* pv. *vesicatoria* (*Xcv*) effector AvrBsT physically interacts with pepper arginine decarboxylase (CaADC1), mediating polyamine metabolism for ROS signaling, cell death, and defense responses in plants (**Table 1**; Kim et al., 2013). *CaADC1* silencing in pepper plants greatly reduces ROS and nitric oxide (NO) bursts, as well as the cell death response during *Xcv* infection, suggesting that arginine decarboxylase is required for polyamine and ROS signaling in the HR cell death response.

## Concluding Remarks

Microbial pathogens have evolved efficient strategies to overcome plant innate immunity for the establishment of compatible plant-pathogen interactions. Despite their evolution over a long period of time, pathogen effectors target a common host protein network, suppressing the common immune system of all plants (Weßling et al., 2014). In particular, adapted pathogens have developed effective weaponry to compete with the host plants and defeat evolving plant immunity (Jones and Dangl, 2006). In this arms race, the most frequent target of pathogen attack is a powerful plant weapon system that inflicts immense damage to invading pathogens in a short period of time (Doehlemann et al., 2014).

Effector targeting toward ROS signaling networks in plants is proposed in **Figure 3**. Adapted pathogens have evolved virulence effectors to inhibit both the generation and accumulation of ROS in the apoplastic space, ultimately leading to the inhibition of the intracellular signaling required for the powerful second ROS burst. Plant pathogens deliver virulence effectors into the apoplastic area and cytoplasm of plant cells. Apoplastic effectors interfere with the perception of PRRs by MAMPs or PAMPs, preventing activation of membrane-bound NADPH oxidase RBOHs. Removal of ROS from the apoplastic area reduces direct toxicity to pathogens and blocks plant cell wall reinforcement, which may be beneficial for the successful intracellular invasion and colonization of microbial pathogens. Cytoplasmic effectors target plant PRRs, the RLCK BIK1, MAPK cascades, and WRKY transcription factors, suppressing the expression of RBOHs and NADP-MEs that are essential for robust ROS generation (**Figure 3**; Singh et al., 2016). During pathogen infection, certain cytoplasmic effectors interfere with vesicle trafficking to suppress the transport of ROS-producing RBOH enzymes to the plasma membrane (Macho and Zipfel, 2015). Secreted pathogen effectors block the metabolic coenzyme NADP-ME, inhibiting the transfer of electrons to NADPH oxidases (RBOHs). However, when specific pathogen effectors interact directly or indirectly with intracellular NLR proteins, these effectors function as avirulence factors to trigger resistance (R) gene-mediated immunity, the so-called ETI (Dangl et al., 2013). The effector-NLR complex leads to a strong apoplastic ROS burst and HR cell death responses. Apoplastic ROS are toxic to pathogens and activate MAPK cascades and NADPH oxidase (RBOH) enzymes. The first phase of ROS production by PTI following PAMP recognition may eventually have a feedback effect on the second phase of ROS production, resulting in a more powerful ETI (**Figure 2**; Yoshioka et al., 2003; Ishihama et al., 2011; Adachi et al., 2015).

Pathogens have developed virulence effectors to circumvent the newly changed plant immune system during the coevolution of both pathogens and host plants. In host plants, effector-targeted host proteins inside the plant cell can be modified to restrict pathogen invasion. Virulence effectors interact with different target host proteins in the plant cell, which can ultimately result in the suppression of ROS production in the apoplastic area. Once NADP-ME fails to supply NADPH to the NADPH oxidase (RBOH) as a result of its mutation, apoplastic ROS production is inhibited and pathogens can overcome plant immunity (Singh et al., 2016). These results suggest that ROS contribute directly to plant immunity. PTI and ETI are closely related and a strong ROS burst is essential for ETI. The metabolic cellular processes related to ROS generation are required to sustain PTI and reinforce the immune response through ETI. The key proteins and/or enzymes involved in ROS production may be supplied by activation of MAPK signaling pathways (Ishihama et al., 2011; Adachi et al., 2015). The biomembrane channels, aquaporins, mediate H_2_O_2_ transport across biological membranes. However, when effectors interfere with MAPK cascades and WRKY transcription factors (Zhang et al., 2007, 2012; Wang et al., 2010; Feng and Zhou, 2012; Le Roux et al., 2015), the second phase ROS burst and accompanying ETI are severely inhibited (Adachi et al., 2015).

Microbial pathogens have evolved versatile effectors to target the cellular processes associated with plant ROS production. Despite recent advances in our knowledge of pathogen effector targets in ROS signaling networks in plants (**Table 1**), the key host factors directly linking ROS signaling to sites of attempted pathogen invasion are not fully understood. Further elucidation of the molecular and cellular functions of pathogen effectors and host factors underlying the ROS-mediated innate immune system will provide important clues to understand better how versatile effectors have evolved to converge on ROS signaling networks in plants.

## Author Contributions

NSJ designed the outline of the manuscript and wrote major part of the story and BKH added his previous bacterial data and expertise to upgrade the whole text and Figures.

## Conflict of Interest Statement

The authors declare that the research was conducted in the absence of any commercial or financial relationships that could be construed as a potential conflict of interest.
